# Molecular Characterization of Rotavirus From Under‐Five Children Presenting With Diarrhea at a Tertiary Hospital in Northeast Nigeria

**DOI:** 10.1155/ijpe/7023953

**Published:** 2026-08-20

**Authors:** Kellu Bukar Ali, Yahaya Yaqub, Mairo Usman Kadaura, Yakubu Mohammed Yakubu, Batula Bishara Daggash, Aishatu Baba Shettima, Nasir Garba Zango, Mohammad Ibrahim Dauda, Zainab Rabilu Daninna, Mohammed Yahaya, Galadima Bala Gadzama, Sambo Bello Zailani

**Affiliations:** ^1^ Department of Medical Microbiology, University of Maiduguri, Maiduguri, Borno State, Nigeria, unimaid.edu.ng; ^2^ Department of Medical Microbiology, Ahmadu Bello University Zaria, Zaria, Kaduna State, Nigeria, abu.edu.ng; ^3^ Department of Medical Microbiology, Federal University of Health Sciences, Azare, Bauchi State, Nigeria; ^4^ Department of Medical Microbiology, University of Maiduguri Teaching Hospital, Maiduguri, Borno State, Nigeria, unimaid.edu.ng; ^5^ Department of Medical Microbiology and Parasitology, Bayero University Kano, Kano, Kano State, Nigeria, buk.edu.ng; ^6^ Department of Medical Microbiology, Usmanu Danfodiyo University Teaching Hospital, Sokoto, Sokoto State, Nigeria, udusok.edu.ng; ^7^ Department of Medical Microbiology, Abubakar Tafawa Balewa University Teaching Hospital, Bauchi, Bauchi State, Nigeria, atbuth.org.ng

**Keywords:** diarrhea, Nigeria, rotavirus, RT-PCR, under-five children, vaccine-preventable diseases

## Abstract

**Background:**

Diarrhea is a leading cause of morbidity and mortality among children in developing countries. Rotavirus is a significant cause of acute watery diarrhea in children under 5 years of age, which can be fatal. This study is aimed at investigating the prevalence of rotavirus gastroenteritis (RVGE) in children and characterizing the prevalent circulating genotypes.

**Material and Methods:**

A cross‐sectional study was conducted, enrolling 173 children under the age of 5 years who presented with acute diarrhea lasting less than 2 weeks at the University of Maiduguri Teaching Hospital between 2017 and 2018. Stool samples were collected and screened for rotavirus antigen using the lateral flow immunochromatographic method. Stool samples that screened positive were further investigated for VP7 (i.e., G type) and VP4 (i.e., P type) rotavirus genotypes using reverse transcription–polymerase chain reaction (RT‐PCR).

**Results:**

The prevalence was determined to be 28% by the immunochromatographic method and 14.5% by RT‐PCR (*N* = 173). The identified G types included G1, G2, G1G2, G9, G12, and G NT (G nontypeable), whereas the P types identified were P4, P6, and P8. The most frequently observed rotavirus genotype combination was G9P[6] 7 (28%), while mixed genotype infection with unusual strain G1G2P[4, 6, 8] 1 (4%) was also detected.

**Conclusion:**

This study reported a high prevalence of RVGE in the northeast region of Nigeria. As the current ROTAVAC in use in routine immunization in Nigeria comes to its third year, it is necessary to evaluate the vaccine efficacy so as to reduce the deaths associated with RVGE in Nigeria.

## 1. Background

Rotaviruses belong to the Sedoreoviridae family [1]. It has a double‐stranded RNA genome that contains 11 segments. Each segment codes for viral proteins (VPs) and nonstructural proteins (NSPs), coded as VP1, VP2, VP3, VP4, VP6, VP7, and NSPs 1–6. The capsid covers the genome and has no envelopes. The VP6 proteins serve as the foundation for the classification of rotaviruses into Groups A–H. Human diseases are caused by Serotypes A, B, and C, whereas other serotypes can cause animal diseases. VP4 and VP7 are used to classify rotaviruses into G and P types, respectively. Neutralizing antibodies are produced against these capsid proteins [2].

Rotaviruses cause diarrhea with complications of dehydration and derangement of electrolytes, which could be fatal if proper management is not instituted. Globally, rotavirus is responsible for an estimated 454,000–705,000 fatalities, predominantly affecting low‐ and middle‐income countries [3]. Cunliffe et al. reported that 85% of deaths associated with rotavirus infections occurred in Africa and Asia [4]. In Nigeria, specific prevalence rates have been reported as 15.6% in Zaria, 13.8% in Jos, and 25.5% in Sokoto [5–7]. A study conducted in southwest Nigeria suggests that rotavirus prevalence could be influenced by environmental factors such as humidity. Specifically, the study observed a higher prevalence rate of 19.3% during the dry season compared to 8.5% during the rainy season [8].

Rotavirus gastroenteritis (RVGE) is a vaccine‐preventable disease associated with high morbidity and mortality worldwide. RVGE is seen mostly in children aged less than 5 years, with higher incidences between the ages of 6 months and 2 years [6]. Adults are less susceptible to the infection, except in those traveling to endemic regions with no prior immunity. Individuals above the age of 65 years, immunocompromised hosts, and caregivers of patients have increased infection rates [9]. Ali et al. reported in the study of which this was part that the likelihood of contracting RVGE was higher in residents with inadequate water supply, especially when there are individuals with RVGE living in the household [10].

RVGE is transmitted via the fecal–oral route, primarily through the consumption of contaminated food and water or through contact with contaminated objects such as toys. The incubation period ranged from 1 to 3 days. Patients typically experience fever, vomiting, stomach cramps, and sudden watery diarrhea. Patients could continue to shed the virus in their stool for weeks, even after the symptoms have subsided. Continued viral shedding in stool is an important route of transmission for RVGE [11]. Vaccines are an important component for reducing the prevalence of RVGE and the severity of the disease. Evidence has shown that combining vaccination and proper hygiene practices reduces morbidity and mortality.

The data generated from this research will provide information on the common circulating viral serotypes in the northeast region of Nigeria. This will provide baseline information that will be important to policymakers in choosing the best‐fit vaccine for Nigeria as well as neighboring countries like Chad, Cameroon, and Niger.

## 2. Material and Methods

### 2.1. Study Area

The investigation was conducted at the University of Maiduguri Teaching Hospital (UMTH) situated in Borno State, in the northeastern region of Nigeria [12]. Most of the population of Borno State is of low socioeconomic status and primary educational level [13]. Anecdotal evidence reveals that around 13,000 patients are admitted each year at UMTH, of whom approximately 500 have diarrhea as the primary presenting complain.

### 2.2. Study Population

The study population comprised children under 5 years of age who were receiving care between 2017 and 2018 at the UMTH, Borno State in northeast Nigeria.

### 2.3. Study Design and Sampling Method

This study employed a cross‐sectional hospital‐based design as stated in an earlier published study [10]. The sample size was calculated as 173 using Fisher′s formula for sample size. A simple random sampling technique was used, wherein a table of random numbers was employed to generate the sample.

### 2.4. Ethical Consideration

The ethical review committee of the UMTH, Nigeria, reviewed and approved this study.

### 2.5. Inclusion Criteria

Children under the age of 5 years who presented with diarrhea, defined as the passage of more than three episodes of watery stools per 24 h, were included in the study.

### 2.6. Exclusion Criteria

Children fitting the inclusion criteria but with chronic diarrhea (i.e., diarrhea lasting more than 2 weeks) and/or those with bloody diarrhea were excluded from the study.

### 2.7. Sample Collection and Storage

Stool samples were collected by instructing parents and caregivers on sample collection methods for outpatients, while for inpatients, sample collection was supervised by nurses. Stool samples (5–10 mL) were collected in a sterile, wide‐mouthed and properly labeled container. Each sample was promptly transported to the laboratory in a reverse cold chain manner and was maintained at 4°C prior to processing.

### 2.8. Screening by Immunochromatographic Method

The initial screening of the samples was done by an immunochromatographic test (i.e., lateral flow immunoassay) by employing the Rota‐dipstick (Mascia Brunelli S.p.a, Milano, Italy). This procedure was performed in accordance with the manufacturer′s instructions. Samples that tested positive were subsequently stored at −20°C for about 6 months before molecular characterization.

### 2.9. Molecular Characterization Method

The dsRNA was extracted using both the phenol/chloroform RNAID kit and the RNAID extraction method [14]. The RNA‐containing supernatant was then meticulously transferred into a sterile Eppendorf tube and kept at −20°C until it was required for reverse transcription–polymerase chain reaction (RT‐PCR) after complete collection of stool samples.

The VP7 gene genotyping was done according to the protocol used by Vera Gouvea/Itturiza–Gomara/Banerjee [15, 16]. Thus, the primer cocktail used included Beg9, end9, RVG9, aBT1, aCTI, aET3, aDT4, aAT8, aFT9, G10, and G12. These primers were used to identify the genotypes G1, G2, G3, G4, G8, G9, G10, and G12, respectively. The Gentsch et al. primer cocktail was used for VP4 genotyping. Thus, the cocktail of primers used included Con2 and Con 3, along with 1T1, 2T1, 3T1, 4T1, and 5T1. These were used to identify the P[8], P[4], P[6], and P[10] genes.

The amplification process involved the following steps separately for the VP7 and VP4 genotypes: an initial denaturation at 94°C for 2 min, followed by 30 cycles consisting of denaturation at 94°C for 1 min, annealing at 42°C for 2 min, and extension at 72°C for 3 min. The process was concluded with a final extension step at 72°C for 5 min.

Electrophoresis was performed using a 1.0% agarose gel. Standard molecular weight markers were used in this study, and a negative control was included. Thus, successful amplification of VP7 and VP4 was performed by RT‐PCR.

## 3. Result

One hundred and seventy‐three (*N* = 173) stool samples from children within the age bracket of 0– 59 months were recruited for the study. Male and female children accounted for 52.6% (91) and 47.4% (82), respectively. The median age of the children was 11 months. The Rota‐dipstick screening kit identified 48 (28%) as positive for rotaviruses, out of which 25 (14.5%) of the samples were positive for RT‐PCR analyses.

**Figure 1:**
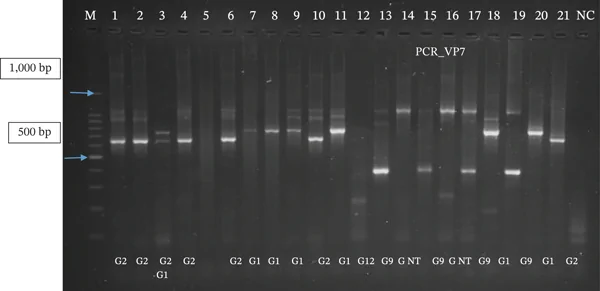
A snapshot of gel electrophoresis of the rotavirus G protein types (i.e., VP7) gene found in this study. Note: M, molecular weight marker; G, glycoprotein type; NC, negative control; NT, nontypeable G strains; bp, base pair.

The identified rotavirus RNA G types were G1, G2, G1G2, G9, G12, and G NT (G nontypeable). The proportions of these are presented in Table 1, while the gel electrophoresis of the G types is shown in Figure 1.

**Table 1:** Frequency table for the different G types detected in the rotavirus RNA in this study.

G type	Frequency (*n*)	Percentage (%)
G1	6	24
G2	6	24
G1G2	1	4
G9	8	32
G12	1	4
G NT	3	12
Total	25	100

**Figure 2:**
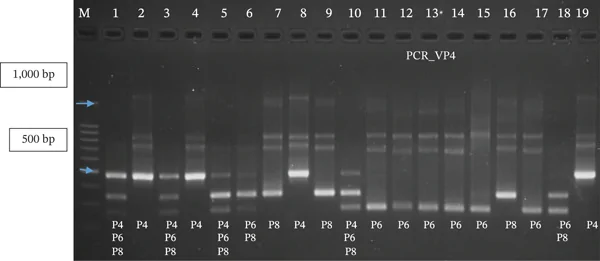
A snapshot of gel electrophoresis of the rotavirus P protein type (i.e., VP4) gene found in this study. Note: P, P protein; M, molecular weight marker; bp, base pair.

The detected P types included P[4], P[6], and P[8]. The number (and proportions) of the P types and combinations are P[4] 5 (20%), P[6] 10 (40%), P[8] 4 (16%), P[6, 8] 2 (8%), P[4, 6, 8] 4 (16%) patients. Mixed infections, with more than one genotype, were observed in this study. Important to note is that Lane 3 of the gel electrophoresis in Figures 1 and 2 shows G1G2P[4, 6, 8]. Five other mixed P types were observed in Lanes 1, 3, 5, 6, 10, and 18, as shown in Figure 2. Although not shown in the gel electrophoresis, there were an additional four G9P[6] and one each of G1P[8] and G2P[4].

**Figure 3:**
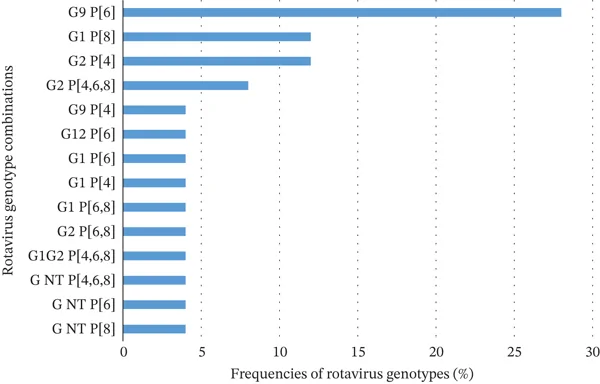
Frequencies of the rotavirus genotypes detected from stool samples in this study.

The rotavirus genotype combinations identified in this study are as shown in Table 2, and the frequencies are illustrated in Figure 3.

**Table 2:** Rotavirus genotype combinations detected in this study.

S/no.	Rotavirus genotype combinations	Number of genotype combinations
1	G NTP[8]	1
2	G NTP[6]	1
3	G NTP[4, 6, 8]	1
4	G1G2P[4, 6, 8]	1
5	G2P[6, 8]	1
6	G1P[6, 8]	1
7	G1P[4]	1
8	G1P[6]	1
9	G12P[6]	1
10	G9P[4]	1
11	G2P[4, 6, 8]	2
12	G2P[4]	3
13	G1P[8]	3
14	G9P[6]	7
	Total	25

## 4. Discussion

The observed prevalence was 14.5% (i.e., by molecular method) in this study, could be considered high as it is around the 15% benchmark, and is similar to most of the northeast states prior to the introduction of the rotavirus vaccine (ROTAVAC) into the routine immunization in Nigeria [17]. A prevaccine introduction study in Maiduguri also reported a higher prevalence of 41% (*N* = 188) compared to that of this study, 28% (*N* = 173), using the same methodology (i.e., by immunochromatographic method) [18]. The observed difference in prevalence in this study between the immunochromatographic method and the molecular method of 28% and 14.5%, respectively, may have been due to loss of viral RNA material because of an inconsistent power supply during storage at −20°C. We could not find similar studies reported from the northeast of Nigeria in the prevaccination period that utilized molecular methodology, so a comparative prevalence could not be ascertained on that basis. However, a study done around the time when this study was being conducted reported no molecular results for rotavirus despite reporting positive findings by the immunochromatographic method [19].

Globally, there are five prevalent strains of rotavirus, namely, G1, G2, G3, G4, and G9. Each of these strains is characterized by the VP4 specificities of P[4], P[6], and P[8] [20]. The most common G type detected in the present study was G9, which is an emerging strain of RVGE and was seen in about 32% of the positive isolates in this study. This was similar to a WHO‐sponsored sentinel surveillance study in six different sites in Nigeriawhich was used to guide the Nigerian government on the choice of the ROTAVAC that was used in the country [21]. The different P types found in this study (i.e., P[4], P[6], and P[8]) are responsible for at least 90% of human‐related RVGE [22]. The Nigerian government′s choice ofROTAVAC, which is a G9P[11]‐based vaccine, and placed it into the routine immunization schedule in August 2022 [21, 22]. Thus, it can be concluded that even though UMTH was not a WHO sentinel site, which were all tertiary health facilities as well, the genotypes found are in agreement with the WHO surveillance.

G1‐ and/or G2‐based combinations were the next most frequently found in this study (see Figure 3). Earlier studies reported from Kano and Lagos revealed more G2 compared to other strain types, which is different from what was found in this study [22]. G1, on the other hand, has been reported as the most prevalent strain in two consecutive years (i.e., 2014 and 2015) in Enugu, southeast Nigeria. The Enugu study, which was a report for 5 years (i.e., 2011–2016), found other G types more prevalent in other years [23]. This change in genotypes (i.e., genetic reassortment) after some period of time has been reported to be occurring due predominantly to in vivo factors rather than environmental ones [20, 24]. A 10‐year review (i.e., 2013–2023) of rotavirus genotypes in the University of Ilorin Teaching Hospital (UITH), Ilorin (i.e., northcentral), Nigeria, revealed the commonest genotype to G1, while G2 was also significant [20, 21, 25]. Cumulatively, G1 and/or G2 constitute more genotype variations in this study, which is quite different from the reports from the southeast and northcentral regions of Nigeria. This becomes significant considering that G1 and/or G2 are not represented in the ROTAVAC vaccine currently in use in Nigeria. Although it is quite early, this recent claim by UITH Ilorin that the most prevalent strain postvaccine introduction is G1P[8] may be an important inflection point to explain the possibility of the eventual realization of case(s) of vaccine escape to watch out for in Nigeria. This is notably so considering that UITH Ilorin is a pivotal sentinel site of the WHO for rotavirus surveillance in Nigeria [25, 26].

The G12 genotype accounted for 4% of the positive stool samples (Figure 3). The Enugu study also reported G12 as the predominant strain for some time. Thus, it could be inferred that the G12 strain is important in Nigeria. Earlier reports from other parts of the world reveal G12 as an important cause of RGVE, which has led to outbreaks in Nepal and Bangladesh [27, 28].

We found several unusual strains in this study, which was no surprise considering that there have been reports of unusual strains, even of animal origin, causing infection in human subjects [29]. Important to note, however, is that we found G2P[6, 8], which may have occurred because of reassortment, andhas also been documented earlier in studies done in Nigeria [30, 31]. G NT strains composed 12% of the G types detected in the positive stool samples of this study. These G NT strains have been reported earlier in the southern parts of Nigeria [23].

Although postvaccine surveillance has not been systematically undertaken at UMTH, a postvaccine introduction (i.e., 2024) study in a similar setting (i.e., a tertiary health facility) but utilizing the immunochromatographic method in the northeast (i.e., Bauchi State, Nigeria) reported a lower prevalence of 20% (*N* = 200) [32]. This finding of almost halving the RVGE postvaccine introduction should be cautiously observed, as other factors such as exclusive breast‐feeding and mothers′ educational status have been reported to give significant protection to children independent of vaccination [33]. Also, anecdotal evidence suggests that states within the northeast region are yet to have an objective surveillance report, which will yield a more holistic picture. Despite the challenges faced by the decade‐long poor political will toward primary healthcare (PHC) in Nigeria, the recent push by the current federal administration through the National Health Sector Renewal Investment Initiative (NHSRII) has seen major revitalization of PHCs [34]. However, critical infrastructures like linking roads as well as basic income are indispensable if the potential to save 100,00 children due to the introduction of ROTAVAC in Nigeria is to be realized by 2029 [17].

## 5. Conclusion

The high prevalence rate found in this study shows that rotavirus is still a significant cause of RVGE in Nigeria. Thus, we make an argument that the Nigerian federal government should push for an interim vaccine efficacy evaluation in 2026. This would ensure the ROTAVAC vaccine has been given at least a 36‐month period for impact. The result of such vaccine efficacy would provide the federal government with objective data to act upon if the trajectory toward the projected effect of the prevention of death in children by ROTAVAC is to be realized by 2029.

## Author Contributions

K.B.A., Y.Y., G.B.G., and S.B.Z. were involved in the research idea, study design, and implementation of the study. K.B.A., Y.Y., Z.R.D., and S.B.Z. were responsible for the analysis and interpretation of data. K.B.A., Y.Y., M.I.D., M.U.K., Y.M.Y., B.B.D., N.G.Z., M.Y., A.B.S., and G.B.G. wrote the main manuscript and prepared the tables and figures. All authors reviewed the manuscript.

## Funding

No funding was received for this manuscript.

## Disclosure

A preprint has previously been published by Ali et al. [35].

## Ethics Statement

Ethical approval in accordance with the Declaration of Helsinki was obtained from the University of Maiduguri Teaching Hospital, Research Ethics Committee in accordance with the Helsinki Declaration.

## Consent

Informed consent (i.e., assent) to participate in the study was obtained from the parents or legal guardians of all the participants (as they were all below the age of 16). This included permission for anonymous use of data (e.g., molecular studies) for the purpose of publication.

## Conflicts of Interest

The authors declare no conflicts of interest.

## Data Availability

All data generated during this study are included.
